# ASSOCIATIONS OF CIRCADIAN REST/ACTIVITY RHYTHMS WITH METABOLIC SYNDROME IN MIDDLE-AGED AND OLDER ADULTS

**DOI:** 10.1093/geroni/igae098.0252

**Published:** 2024-12-31

**Authors:** Yiwei Yue, Jill Rabinowitz, Yang An, Marc Kaizi-Lutu, Chunyu Liu, Eleanor Simonsick, Jennifer Schrack, Adam Spira

**Affiliations:** Johns Hopkins University, Baltimore, Maryland, United States; Rutgers, The State University of New Jersey, New Brunswick, New Jersey, United States; National Institute on Aging, Baltimore, Maryland, United States; Johns Hopkins University, Baltimore, Maryland, United States; Johns Hopkins University, Baltimore, Maryland, United States; National Institute on Aging, Baltimore City 0400 Baltimore City, Maryland, United States; Johns Hopkins Bloomberg School of Public Health, Baltimore, Maryland, United States; Johns Hopkins University, Baltimore, Maryland, United States

## Abstract

Circadian rhythm disturbances often co-occur with adverse cardiometabolic outcomes, including high blood pressure, obesity, and diabetes. However, the links between disrupted circadian rhythms and metabolic syndrome (MetS) are understudied in later life. This study examined associations between actigraphic circadian rest/activity rhythm (CRAR) metrics and MetS in 388 Baltimore Longitudinal Study of Aging participants who completed 6.6±1.0 nights of wrist actigraphy (Mean age=72.6±10.1, 20.6% Black adults, 54.1% women) and measures of MetS. Predictors were relative amplitude (RA), interdaily stability, intradaily variability, midpoint of total activity during the least active continuous 5 hours, and midpoint of total activity during the most active continuous 10 hours (M10 time). Participants were classified as having MetS if they had ≥3 of the following: triglycerides >150 mg/dL; fasting blood glucose ≥100 mg/dL; high-density lipoproteins <40 mg/dL for men and <50 mg/dL for women; blood pressure ≥130/85 mmHg; waist circumference ≥102 cm for men and ≥88 cm for women. Overall, 39 participants had MetS at baseline and 24 developed MetS over 4.5±2.0 years of follow-up. Adjusting for demographics, physical activity, depressive symptoms, time, and interactions of time with covariates, higher RA was cross-sectionally associated with 29% lower odds of MetS (per SD, B=0.71, p=0.046). Longitudinally, a later M10 time was associated with a faster rate of increase in the odds of developing MetS (per SD, B=1.14, p=0.014). Findings link weaker and more phase-delayed CRARs to higher odds of MetS in middle-aged and older adults. Studies are needed to identify mechanisms linking disrupted CRARs to metabolic disorders.

